# Photoluminescent Detection of Dissolved Underwater Trace Explosives

**DOI:** 10.1100/tsw.2010.41

**Published:** 2010-04-01

**Authors:** Tye Langston

**Affiliations:** Department of Ocean Engineering, Florida Atlantic University, Boca Raton, FL, USA

**Keywords:** explosives, explosive detection, nitroglycerin, europium, thenoyltrifluoroacetone, 10-phenanthroline, lanthanide, coastal security

## Abstract

A portable, rapid, and economical method for *in situ* trace explosive detection in aqueous solutions was demonstrated using photoluminescence. Using europium/thenoyltrifluoroacetone as the reagent, dissolved nitroglycerin was fluorescently tagged and detected in seawater solutions without sample preparation, drying, or preconcentration. The chemical method was developed in a laboratory setting and demonstrated in a flow-through configuration using lightweight, inexpensive, commercial components by directly injecting the reagents into a continually flowing seawater stream using a small amount of organic solvent (approximately 8% of the total solution). Europium's vulnerability to vibrational fluorescence quenching by water provided the mode of detection. Without nitroglycerin in the seawater solution, the reagent's fluorescence was quenched, but when dissolved nitroglycerin was present, it displaced the water molecules from the europium/thenoyltrifluoroacetone compound and restored fluorescence. This effort focused on developing a seawater sensor, but performance comparisons were made to freshwater. The method was found to perform better in freshwater and it was shown that certain seawater constituents (such as calcium) have an adverse impact. However, the concentrations of these constituents are not expected to vary significantly from the natural seawater used herein.

### INTRODUCTION

The terrorism threat to the U.S. and other countries is as severe as ever and constantly changing. Accordingly, the evolving tactics of terrorists must be evaluated and met with improved counterterrorism efforts. Explosive materials form the heart of many terrorism plots and their detection is a crucial part of this effort. Furthermore, abandoned explosives (such as munitions from previous wars, accidents, or military testing) create environmental hazards. Explosives can exist in bulk or trace amounts, as well as in dry or wet environments. There are several approaches to detecting bulk explosives in both dry and wet environments, as well as trace explosives in dry environments[1,2,3]. However, aqueous trace explosive detection is an area in need of improvement, specifically in regard to seawater. Dissolved trace explosives may occur in a number of aqueous environments, such as in contained water solutions, coastal and port

*Current address: Science and Technology Department, Naval Surface Warfare Center, Panama City, FL
©2010 with author.
Published by TheScientificWorld; www.thescientificworld.com environments, or offshore areas, presenting environmental and security risks[4,5]. In addition to airports and land borders, terrorism threatens coastal boundaries as ports, tunnels, and numerous waterways are also vulnerable to explosive transport and attack. In addition to aiding environmental analysis and cleanup, underwater detection of explosive traces helps to close the security gap in marine environments, just as dry screening for explosives helps to maintain airport security. Many of the current aqueous trace explosive detection methods are constrained to laboratories, focus on low-salt groundwater, require extensive sample preparation, take a significant amount of time to complete, and are prohibitive in equipment cost[6,7,8,9]. The identification of a fast, portable, inexpensive, water-borne trace explosive detection method that could be applied to both fresh- and saltwater would serve the goals of security, forensic analysis, and environmental remediation.

The traditional means of analyzing aqueous samples for trace explosives is detailed by the U.S. Environmental Protection Agency (US EPA) protocol SW-846 Method 8330, which requires on-site sampling of contaminated groundwater followed by transportation to a laboratory for analysis[10]. This method, which is capable of ppb detection levels, calls for two separations using reversed-phase high-pressure liquid chromatography (HPLC) with UV detection. The first separation typically takes 30–40 min[11] and the second, confirmatory analysis can be achieved within 15 min[10,11]. Aqueous samples of low concentration are extracted with a salting-out procedure that includes sodium chloride and an organic solvent. Although the HPLC method is sensitive and the actual experimental work can be accomplished in about an hour, this method is suited only for the laboratory and rapid field results cannot be attained. Samples must be transported to a laboratory for analysis and in emergencies, days can be expected, while it may take months in routine situations[9].

Other methods have been developed that provide fast, accurate results, such as the microchip-based approaches described by Wallenborg and Bailey[11] and Hilmi and Luong[12]. However, while these methods may be adapted to field use eventually, they are currently described as laboratory procedures and the samples often require time-consuming pretreatment, dissolving them in appropriate buffer solutions before analysis[11,12]. Wallenborg and Bailey's approach utilized indirect laser-induced fluorescence to detect various nitroaromatics and nitramines after separation by micellar electrokinetic chromatography[11]. They used a 750-nm near-infrared diode laser for excitation and added Cy7 dye to the running buffer as a visualizing agent, which they detected with a photomultiplier tube. After extraction from soil, they were able to identify 1-ppm amounts of nine nitroaromatic compounds, including TNT and tetryl, but could only detect nitramines (RDX and HMX) at much higher concentrations (>2000 ppm)[11]. Hilmi and Luong also demonstrated an electrochemical chip-based detector by using capillary electrophoreses to detect TNT. Applying amperometric detection with a gold electrode, they were able to identify TNT at 100–200 ppb in less than 4 min[12].

Explosive-detecting colorimetry is based on the formation of colored products upon reaction of nitroaromatic and nitramine compounds with alkali or acidic solutions[13]. Although it is not immediately applicable to dissolved trace explosives in aqueous solutions, it has been applied to fresh- and seawater solutions by separating the explosives out. In an effort to predict how long trace explosives could be detected on aircraft materials in marine environments, Kamyshny et al. evaluated their persistence on polyethylene, glass, aluminum, and seat fabric after exposure to tap water, artificial seawater, and natural seawater[14]. After immersion, the material samples were dried and tested for explosives using a commercial colorimetric testing kit. By pipetting the colorimetric reagents directly onto the different materials and visually evaluating the color changes, they qualitatively detected TNT, RDX, Semtex, and PETN. Jenkins et al.[15] and Craig et al.[13] also applied colorimetry to analyze TNT and RDX dissolved in water. They first recovered explosive traces by filtering 2-l volume samples through two membranes and then extracted the explosives from the membranes using acetone. The colorimetric reagents were added to the acetone/trace explosive solution, which was then analyzed in a spectrophotometer. Kamyshny et al. noted that approximately 10 min was optimal for color development in their experiments, after which samples without explosives also began to develop color (false-positives). Similarly, a 15-min incubation period is recommended for the colorimetric system that Jenkins et al. and Craig et al. used[16]. Colorimetric methods provide a useful screening tool and are relatively fast, but the need to extract the trace explosives from water samples before analysis hinders their usefulness for real-time marine field use.

Immunoassays have emerged as a promising technology for rapid, field detection of aqueous trace explosives. They utilize competitive binding reactions in which trace explosive compounds compete with and displace labeled antigens on antibody-coated surfaces. The labeled reporter molecules provide detectable signals (enzymes, chromophores, fluorophores, luminescent compounds, etc.)[17] and explosive detection is accomplished either by measuring the signal reduction on the solid surface or by detecting the displaced labeled molecules free-flowing at a downstream location. With an immunoassay-based flowing sensor, Shriver-Lake et al. reported a TNT sensitivity of 1–10 ppb in a 25% seawater solution[9]. In this case, they detected displaced labeled antigens, which provided a directly proportional indication of the amount of explosive present. In another approach, optical fibers were used as the substrate and the focus was shifted to measuring the reduction in the antigen signal on the fibers instead of looking for displaced free-flowing antigen molecules[18,19,20,21,22,23]. The optical fibers provided the substrate as well as the fluorescence excitation. TNT exposure displaced some of the labeled reporter antigens, which led to a reduction in signal, providing an inversely proportional relationship between the TNT concentration and fluorescence output. Excellent trace explosive detection results have been attained with immunoassays, but they are faulted by a limited life. The presence of trace explosives gradually eliminates the amount of labeled antigens available for continued detection, lowering the detectable signal and eventually requiring replacement of the antibody/antigen-coated surface. Furthermore, the labeled reporter molecules are subject to various forms of degradation (biological, photobleaching, etc.) that naturally reduce the signal and falsely indicate the presence of explosives.

Although several methods exist for detecting water-borne trace explosives, there is still work to be done in creating an economical, portable, and rapid method that can be used in the field for both fresh- and saltwater analysis. HPLC and electrochemical methods provide excellent results in the laboratory, but are not yet portable enough for field use[9,10,11,12]. Most existing test methods, such as HPLC, electrochemistry, and colorimetry, also require significant sample preparation. The electrochemical methods discussed require the use of buffer solutions[11,12] and although the continuous-flow immunosensor reported by Shriver-Lake et al. was able to analyze seawater, it also required a large amount of solvent, with seawater only comprising 25% of the total solution[9]. Colorimetric methods, while portable, require that the sample is either dried or filtered, and extracted with a solvent before analysis[14,16]. And finally, most of the detection methods cannot be operated continuously, such as colorimetry and HPLC, which are conducted on individual samples. Immunoassay sensors have shown good results, but they gradually lose their ability to detect with continued exposure to trace explosives and the environment. The aim of this work is to explore another means of underwater trace explosive detection that may overcome some of these obstacles. Photoluminescent lanthanide elements, such as europium, have previously been utilized for trace explosive detection in dry environments[2,3]. Although such lanthanides fluoresce with high intensity and long lifetimes, they are also subject to severe quenching by water. It is this quenching behavior, in addition to their ability to bond to explosive compounds, that makes them potential indicators of water-borne explosives. For dry analysis, Menzel et al. identified a europium-based photoluminescent method that provides the field utility of colorimetric test kits, but with improved sensitivity[2,3]. In this method, a fluorescent compound is used, which fluoresces when attached to an explosive compound, and loses its fluorescence when not attached to a trace explosive and in the presence of water. Testing is accomplished in a specific order by first concentrating the explosive traces on a surface (filter paper), applying the reagents in an organic solution, and then lastly, rinsing with water. The explosive molecule prevents reagent quenching when attached prior to water exposure. This method is not immediately applicable to aqueous environments because water is applied to the reagents before they can bond to the explosive traces. Statistically speaking, most of the fluorescent compounds will encounter water molecules before explosive molecules in an aqueous solution, creating a fundamentally different situation. Furthermore, the explosive traces are dispersed in a dilute solution instead of concentrated on a filter paper, as they frequently are in dry analyses[2,3]. Therefore, in order to change the method to suit aqueous solution explosive detection, more information is needed on the nature of the bonding. The question becomes: Do the trace explosives only prevent quenching by protecting the fluorescent molecule from water after they are already attached, or can they overcome the quenching effect of hydrated fluorescent molecules? The explosive trace must be able to assure fluorescence in a previously hydrated and quenched fluorescent compound in order to extend this type of photoluminescent detection to aqueous environments.

In the current study, the dry photoluminescent trace explosive detection method described by Menzel et al. is adapted for use in aqueous environments and a continuous-flow sensor prototype is demonstrated. By evaluating the explosive/reagent/water reactions in a free-flowing configuration, a better understanding of how these compounds interact is developed and applied to design an aqueous detection method. Different chemical combinations were considered and quantified in terms of trace explosive detectability, emission, and excitation characteristics. The effects of the added constituents of seawater were also considered in comparison to distilled water, as well as the relative amount of organic solvent used to deliver the reagents.

### PHOTOLUMINESCENT METHOD

The photoluminescent method of explosives detection centers on the lanthanide series of rare earth elements. Some of these elements, such as cerium, terbium, and europium, have a highly fluorescent character, which provides a detectable indication of their presence[24]. However, in order to fulfill their fluorescence potential, they must be sensitized. Lanthanide elements do not absorb energy well, and low levels of energy means low levels of energy out. Sensitization consists of attaching energy-absorbent ligands to the lanthanide ion, which absorb excitation energy and transfer it to the lanthanide element, allowing fluorescence to occur. The sensitizing ligands are typically organic[25]. They act as energy "antennae" to the lanthanide ion and are referred to as "donors", while the lanthanide element that receives the transferred energy is known as the "acceptor". This process is referred to as fluorescence resonance energy transfer (FRET). Förster has shown that the efficiency ($E$) of the FRET process depends on the inverse sixth distance between the donor and acceptor, as shown in Eq. 1[26].

$$E = \frac{Ro^6}{\left(Ro^6 + r^6\right)} \tag{1}$$

where $E$, $Ro$, and $r$ represent the FRET efficiency, Förster distance, and the actual distance between the donor and acceptor, respectively. The Förster distance, $Ro$, is the distance at which energy transfer is 50% efficient and depends on several factors[26].

$$Ro = 9.78\left(10^3\right)\cdot\left(n^{-4}\cdot fd\cdot k^2\cdot J\right)^{\frac{1}{6}}\ \mathring{A} \tag{2}$$

where $n$, $fd$, $k^2$, and $J$ represent the refractive index of the solution, the fluorescence quantum yield of the donor without the acceptor, the dipole angular orientation of the molecules, and the spectral overlap integral of the donor and acceptor, respectively.

There must be a sufficient spectral overlap between the donor emission frequency and the acceptor excitation frequency for FRET to occur. Radiation is first absorbed at the donor's excitation wavelength and then transferred to the acceptor at the donor's emission wavelength (which coincides with the acceptor's excitation wavelength).

An important characteristic of europium, in particular, is that it also exhibits a tendency to lose its fluorescence in the presence of water[27]. The hydrogen bonding forces between water molecules cause them to vibrate and these vibrational overtones act to deactivate the europium fluorescence process[27,28,29]. Lanthanide ions are cationic and it is the dipolar nature of water that draws them to the lanthanides. The negatively charged oxygen of a water molecule is drawn to the positively charged lanthanide. However, there is an exception to this quenching behavior. When explosive traces are bonded to the sensitized europium compound first, they have been shown to make the compound immune to fluorescence quenching by water[2,3]. How the explosive traces protect the europium compound from quenching is not completely known, but it is believed that the explosive trace bonds with the compound and excludes water molecules. Europium(III) is ninefold coordinating and it is typically sensitized with bidentate ligands. It is believed that each bidentate ligand occupies two of the nine sites. Therefore, when four ligands are attached, eight sites are filled and one is left open. When no explosive material is present, water fills this ninth coordination site and quenches the compound, but if a trace explosive occupies this site, the compound becomes hydrophobic[2,3].

Therefore, the photoluminescent method of detecting trace explosives is typically implemented by first tagging the explosive traces with the sensitized lanthanide compounds. This is done by applying sensitized lanthanide ions in a nonwater solution to a dry sample that contains trace explosives. Next, the mixture is rinsed with water to eliminate fluorescence from all compounds that did not previously bond to an explosive compound. This is followed by excitation with a high-energy light source such as UV radiation and measurement of the resulting fluorescence. The fluorescent compounds that bond to the explosive traces retain fluorescence and those that do not achieve this bond are quenched by water[2,3].

### MATERIALS

Nitroglycerin ($C_3H_5N_3O_9$) (NG) was used as the explosive material because of its chemical structure and availability. NG bears nitrites, which are common to many important types of explosives, and are likely the identifiable moieties of these compounds with the method described herein. Nitrite similarity exists between a large family of explosive compounds, including important types such as RDX, Semtex, HMX, PETN, EGDN, nitrocellulose, tetryl, and Smokeless Powder[30]. Assuming that the nitrite moiety of NG is the detectable part, the identification of NG suggests the detectability of several other explosive types. Additionally, NG was chosen because it is widely available due to its common use in the medical field to relax vascular smooth muscles. With regard to the chemical reagents, europium(III) chloride hexahydrate ($EuCl_3 \cdot 6H_2O$) (Eu) was used as the fluorescent lanthanide element, thenoyltrifluoroacetone ($C_8H_5F_3O_2S$) (TTA) and 1,10-phenanthroline ($C_{12}H_8N_2 \cdot H_2O$) (OP) were evaluated as sensitizing ligands, and methanol was used as the reagent solvent. All chemicals were purchased from Sigma Aldrich, with the exception of the NG, which was obtained from a medical supplier. Natural seawater was pumped into the laboratory from approximately 100 yards offshore of the southern Florida Atlantic coast and cycled through a collection tank before being returned. This allowed fresh seawater samples to be obtained at temperatures near that of the ocean, which ranged from 23 to 28$^{o}$C.

### LABORATORY CHEMICAL DETECTION METHOD DEVELOPMENT

A Perkin-Elmer LS50B luminescence spectrometer was used to evaluate the excitation and emission characteristics of the different reagent compounds, as well as their ability to detect NG. Due to their previous use as sensitizing ligands to Eu in dry trace explosives and fingerprint analysis[2,3], both TTA and OP were evaluated for aqueous application. When only one sensitizing ligand was used, it was mixed with the Eu in a 5:1 ratio. This ratio was chosen because the Eu ion accepts nine bonds and both TTA and OP are bidentate ligands[3]. Each bidentate ligand can fill two bonding sites, allowing only four of them to react with each Eu ion. Increasing the ratio to 5:1 provides a slight excess that assures that full coordination is achieved. For the same reason, a mixing ratio of 3:3:1 was used when both TTA and OP were used, and they were evaluated using both possible mixing orders. Because the goal of this effort was to create a seawater sensor, most of the experiments were conducted with seawater, instead of freshwater.

The first step taken to evaluate the different compounds was to examine their excitation and emission characteristics, which must be known in order to design an effective fluorescence sensor. Each compound was analyzed with and without the presence of NG. Optimal excitation was determined by scanning the excitation wavelengths from 200 to 800 nm, and recording which excitation wavelength resulted in the greatest intensity emission near Eu's characteristic wavelength (613 nm). Once optimal excitation wavelengths were determined for the different compounds, they were then used to excite the compounds and measure the exact emission characteristics. The characteristic emission wavelength of Eu alone is known to be 613 nm[27], but it is important to know if this wavelength is prone to shifting upon reaction with the sensitizing ligands, methanol, NG, or seawater.

The most important attribute of a fluorescence detection method is that it provides a signal differential between solutions that contain trace explosives and those that do not. A large differential is much more important than overall fluorescence intensity. With this focus, each reagent compound was analyzed with and without NG to determine its trace explosive detecting ability. The NG concentrations were kept constant (0 or $10^{-3}$ *M*) and the fluorescent compounds were evaluated through a range of concentrations ($1.25 \times 10^{-5}$ to $10^{-4}$ *M*) to determine which one, at which concentration, provided the largest difference in emitted light intensity between solutions with and without NG.

Once an optimal reagent compound and concentration was identified, the next step was to evaluate the effect of the reagent delivery solvent (methanol). Although it comprised a relatively low proportion of the total solution, it was, nevertheless, a component and it is therefore useful to know how it affects the chemical method. Therefore, while maintaining the concentrations of the reagents and NG constant ($7.9 \times 10^{-5}$ and $10^{-3}$ *M*, respectively), the methanol percentage was varied from 1 to 35%, and the fluorescence differential between NG-laden and NG-absent solutions was evaluated to find an optimal methanol proportion range.

Thus having identified an optimal reagent compound, reagent concentration, and solvent proportion, these variables were then kept constant and the NG concentration in seawater was varied to determine a detection limit. It is important to remember that this detection limit is related to the sensitivity of the laboratory luminescence spectrometer that was used to characterize the chemical method and does not apply to the fluorometer used in the prototype sensor. Any time the optical analysis equipment is changed, its unique design may lead to a different detection limit. Although the detection limit obtained in the lab is not directly related to that of a field-deployable detector, it provided both insight into the behavior of the detection scheme and a starting point in the later determination of the appropriate portable sensor reagent concentration.

Finally, the effects of the various seawater constituents on the detection method were examined. The overarching goal of this effort was to develop a seawater sensor, which drove the use of seawater in the experiments instead of freshwater. While this serves sensor-development efforts, it also introduces some unknowns in the way of seawater constituents. Seawater contains various ions and pH differences that could affect the chemical reagents and NG. Indeed, low pH and the presence of additional metal ions have been reported to affect the fluorescence of lanthanide complexes negatively[29,31,32,33]. The pH effect has been noted to occur at levels lower than approximately 4 or 5[29,31,32], which is outside of typical seawater pH ranges (7.4–8.35)[34,35,36]. The metal ion effects are particular to alkaline earth metal ions, which are known to support metal-exchange reactions with lanthanide ions, leaving them without sensitizing ligands and prone to water quenching. Specifically, this effect has been noted with calcium, but magnesium is another member of this group that can be found in seawater[37]. General tests were conducted to examine the NG detectability differences between fresh- and seawater. In these tests, the NG concentration was kept constant (0 or $10^{-3}$ *M*) and the Eu reagent complex concentration was varied. Although it may first appear backwards and that it would be more appropriate to use a fixed reagent concentration in both sea- and freshwater, this was not possible. The sea- and freshwater solutions required different concentrations of the reagent in order to provide appropriate fluorescence levels for the detection equipment. Too much reagent saturated the spectrometer and too little provided minimal detectable signal. Next, extending beyond the general freshwater/seawater comparisons, the individual influences of calcium ions and acid were more closely examined. Tests were conducted to confirm their potential adverse effects on the Eu compounds, but these effects were not stringently quantified because the pH and earth metal ion levels are not expected to vary considerably from that of the natural seawater used for experimentation herein. The confirmation of these effects is merely to offer an explanation for any detectability differences observed between water and seawater solutions. The acid and calcium ion solutions were obtained by mixing HCl and $CaCl_2$ into distilled water. A NaCl solution was also used as a control to show the inertness of the Cl ion with respect to Eu, as well as to illustrate the detrimental affects of the Ca ions in comparison to the Na ions. The concentrations for each solution were calculated to match the chloride ion concentration with that of the ocean (19.35 g/kg of seawater[37]) and to maintain it as constant across all three solutions, thus eliminating it as a variable. The only drawback of maintaining the chloride ion constant was that the concentration of the calcium ion was half that of the sodium and hydrogen ions. Despite this irregularity, the results proved meaningful.

### PROTOTYPE SENSOR

To demonstrate the viability of the detection method in a rapid, inexpensive, and lightweight package, a benchtop flow-through sensor was constructed and operated. A fluorometer and a static mixer constituted the primary components. The fluorometer used (WETStar by Wet Labs.) was initially designed for chlorophyll detection and therefore had to be modified for the excitation and emission characteristics of the Eu-based reagents. A custom LED was installed to provide excitation centered at 370 nm and an optical filter was used that allowed for the detection of Eu's characteristic emission wavelength (613 nm). The optical filter had a transmittance peak near 613 nm, with a half-peak bandwidth of approximately 20 nm.

The selection of 370 nm as the excitation wavelength was a practical and economical decision, keeping the design of a field-deployable sensor in mind. As is pointed out later in this document, the ideal excitation wavelengths for the two sensitizing ligands were not exactly 370 nm, but this wavelength was chosen because it is acceptable and commonly available in a standard LED. The impact of shifting the excitation wavelength was shown to be minimal through luminescence spectrometry[38], and although the LED emission is centered at 370 nm, its output covers a spectral range that brings it closer to the compound's ideal excitation wavelength.

Low flow rates through the sensor are desirable so as to not waste the reagent solution, but at the same time, good mixing is a prerequisite for the detection method. Therefore, a static mixer was employed upstream of the sensor so both needs could be met. Having no moving parts, static mixers are a reliable, economical way to achieve mixing, even in laminar fluid flow conditions. Using a series of fixed elements, they repeatedly divide and recombine the fluid stream. In this case, a 24-element static mixer was used and the solutions were passed through the fluorometer at an average flow velocity of 0.1 m/sec. Furthermore, during fluorometer calibration, it was discovered that under high NG concentrations, a longer period of time was required for the reactions to complete. At low NG concentrations, the reactions completed instantaneously. To account for this effect and allow extra reaction time, an additional length of tube (2.4 m) was included between the static mixer and the fluorometer. Fig. 1 provides a schematic of the benchtop sensor setup.

Before proceeding to flowing solutions, the sensor was calibrated with static blanks of seawater/reagent/NG mixtures to limit the amount of chemicals used. Calibration was necessary because the fluorometer has a different sensitivity than the luminescence spectrometer. It was important to identify an appropriate reagent concentration for the fluorometer so the flowing test could be correctly planned and conducted. Several reagent concentrations were evaluated in order to select a concentration that could identify a broad range of NG concentrations using the fluorometer. Focus was placed on selecting a concentration that provided a strong signal, but did not saturate the fluorometer. Then, after determining an appropriate reagent concentration, the NG concentration was varied and the sensor output signal was measured to calibrate the sensor.

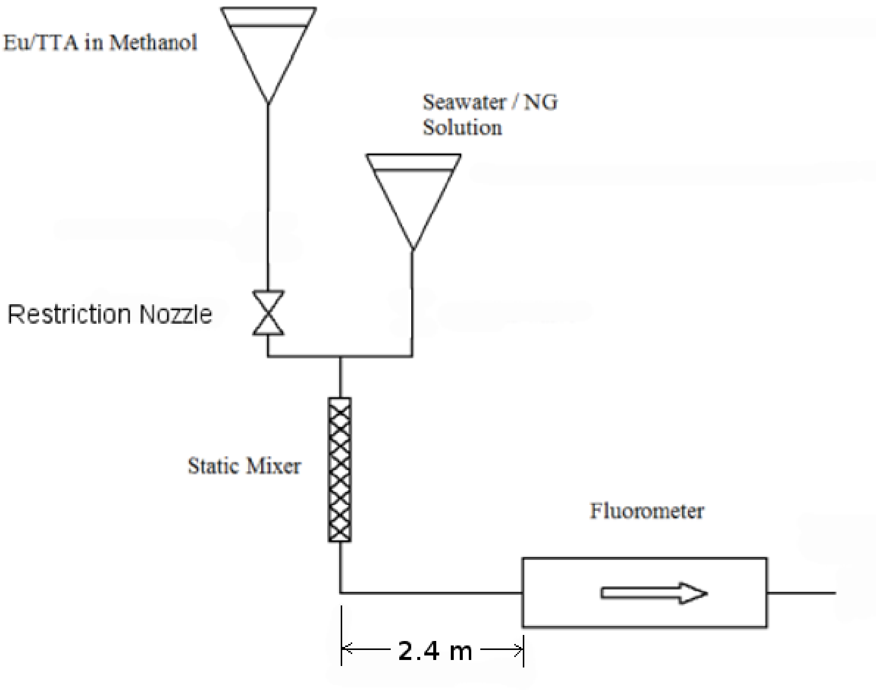

**FIGURE 1.** Fluorometer flowing setup schematic.

## RESULTS

### Emission and Excitation Characteristics

Luminescence spectrometric analysis verified that the emission wavelength for all of the Eu compounds analyzed remained constant. Minimal, if any, shifting of Eu's characteristic emission wavelength (613 nm) occurred with the addition of OP, TTA, NG, methanol, and seawater to the Eu ion.

Similarly, the excitation wavelengths depended strictly on the characteristic excitation wavelengths of the sensitizing ligands and did not appear to shift upon exposure to the other reagents. When TTA was used, an optimal excitation wavelength of 382 nm was found and when OP was used, the optimal excitation was identified at 310 nm. When both ligands were used together, excitation peaks occurred at both wavelengths. Based on these results, the use of OP alone as a sensitizing ligand was discontinued because of its short-wavelength excitation requirements. The overall goal of this research was to identify a practical means of identifying explosive traces in aqueous solutions, and such a short wavelength excitation requirement is not practical because it is not as easily acquired from a commercially available LED.

### Detection Compound Selection

After discovering the short-wavelength excitation requirement of OP, it was eliminated as a lone sensitizing ligand. However, it was included with TTA in some cases to determine if it affected the Eu/TTA compound. When combined with Eu/TTA, OP has been shown to enhance fluorescence synergistically[31]. Thus, Eu/TTA, Eu/TTA/OP, and Eu/OP/TTA were evaluated. Eu/TTA/OP and Eu/OP/TTA are identical, except the mixing order of TTA and OP was reversed. Fluorescent compound comparison testing in the luminescence spectrometer yielded the results shown in Fig. 2.

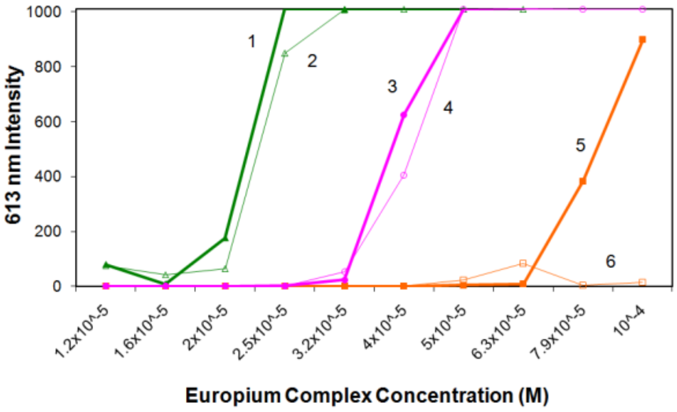

**FIGURE 2**. A 613-nm emission intensity in seawater of (1) Eu/TTA/OP with NG ($10^{-3}$ *M*), (2) Eu/TTA/OP without NG, (3) Eu/OP/TTA with NG ($10^{-3}$ *M*), (4) Eu/OP/TTA without NG, (5) Eu/TTA with NG ($10^{-3}$ *M*), and (6) Eu/TTA without NG. The reagent solvent (methanol) comprised 5% of the solution.

Upon review of Fig. 2, it becomes apparent that there are only narrow ranges in Eu/OP/TTA and Eu/TTA/OP concentrations that fall within the luminescence spectrometer's detection limits. There is a quick jump from virtually undetectable fluorescence to too much fluorescence through a relatively small change in Eu complex concentration. This sharp increase in intensity also occurs for the Eu/TTA compound in the presence of NG. However, the Eu/TTA solution without NG did not fluoresce intensely at any concentration tested. Much less fluorescence was observed when NG was absent than when it was present, indicating that this compound is capable of identifying NG in aqueous solutions.

A luminescence spectrometer is not necessary to observe the fluorescence difference between Eu/TTA solutions with and without NG. It can readily be seen with the application of UV light. Fig. 3 illustrates the fluorescence difference between Eu/TTA solutions in seawater with and without NG, illuminated with a handheld UV light (Amberica West, 370 nm) and recorded through photography.

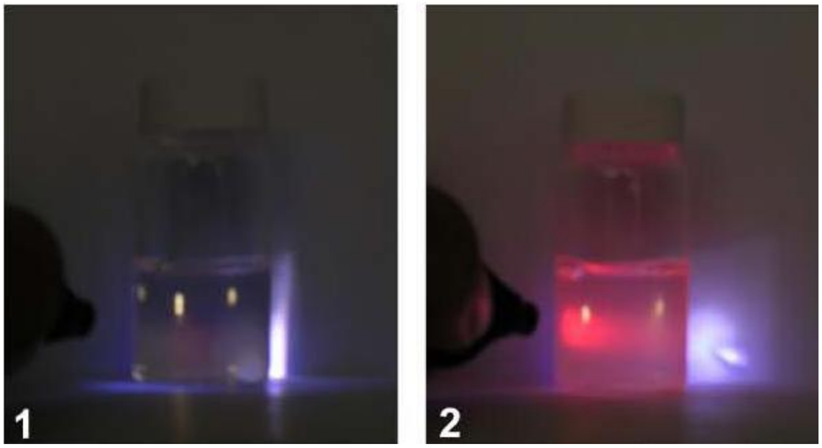

**FIGURE 3**. Eu/TTA solution ($1.4 \times 10^{-3}$ *M*) in seawater (1) without NG and (2) with NG ($5 \times 10^{-4}$ *M*), illuminated by a handheld UV light (370 nm). The reagent solvent (methanol) comprised 5% of the solution.

An additional point of interest that Fig. 2 brings to light is that each compound begins to intensify at a different concentration. Eu/TTA/OP and Eu/OP/TTA behave differently even though they contain the exact same chemicals in the same ratios. Apparently, mixing order (reaction order) of the sensitizing ligands plays a role in the final compound.

### Solvent Volume Percentage Effect

The amount of reagent solvent (methanol) used significantly affected the detection method by altering both the clarity of the solution and the resulting fluorescence output. Having previously ruled out OP compounds, methanol affect studies were conducted using Eu/TTA only. Fig. 4 shows the resulting fluorescence levels for NG-laden and NG-absent solutions as a function of varying methanol content. It can be seen that increasing the percentage of methanol narrows the difference in fluorescence intensity between solutions with and without NG, adversely affecting the NG detection ability.

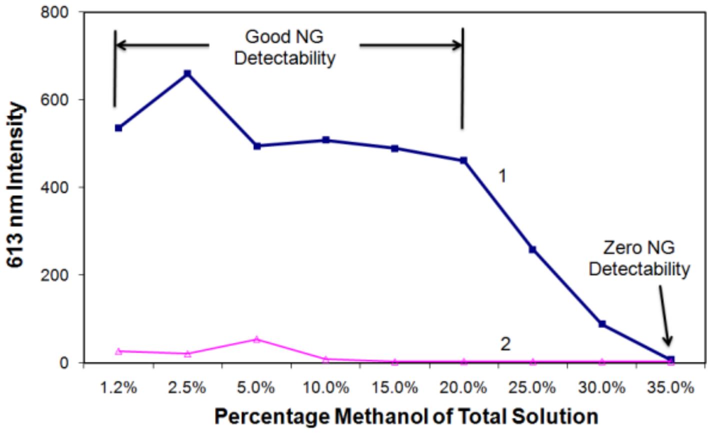

**FIGURE 4**. Eu/TTA 613-nm emission as a function of methanol percentage (1) with NG ($10^{-3}$ *M*) and (2) without NG.

Through photography, it was also observed that a general reduction in solution clarity arose with increased methanol volume percentage when NG was present in solution. Without NG, this effect was minimal. These photographic images are presented elsewhere[38]. Methanol is less polar than water and it is possible that high methanol contents allowed the dissolved NG to collect together instead of dispersing, resulting in a cloudy solution. Solution clarity impacts fluorescence measurement by causing light scattering, thereby compromising the precision of the fluorescence measurement instrument. In addition to solution cloudiness, grouping of the NG compounds would also make them less available to react with the fluorescent compounds and lead to losses in NG detectability.

### Luminescence Spectrometer NG Detection Limit in Seawater

Using the luminescence spectrometer, this chemical method identified NG in seawater solutions as diluted as approximately $4.88 \times 10^{-7}$ *M* (56 ppb), which can be seen in Fig. 5. At relatively high NG concentrations, the emission intensity was found to be strong, but somewhat erratic. This may be due to a reaction speed factor that was not realized until later. At a subsequent time, this effect was noticed during fluorometer testing when the signal was found to oscillate for a short time period (<3 min) with higher NG

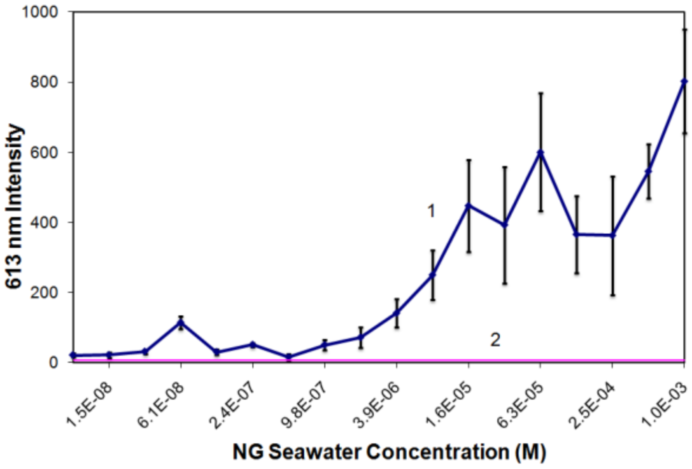

**FIGURE 5**. Eu/TTA 613-nm emission (1) with varying amounts of NG and (2) without NG. The reagent solvent (methanol) comprised 5% of the solution.

concentrations before stabilizing. In light of these observations, it may also be true that the luminescence spectrometer curve could have been smoothed with additional reaction times.

### Effect of Seawater Constituents

By varying the amount of reagent used and keeping the NG concentration constant ($10^{-3}$ *M*), it was shown that the detection method performed better in freshwater than it did in seawater. An equal concentration of NG could be identified at a lower Eu complex concentration in freshwater. Additionally, in seawater, the change from minimal to maximal fluorescence of an explosive-laden solution occurred over a short range in Eu complex concentration. In freshwater, this change occurred over a wider concentration range that began at a much more dilute level. These comparisons can be viewed in Fig. 6.

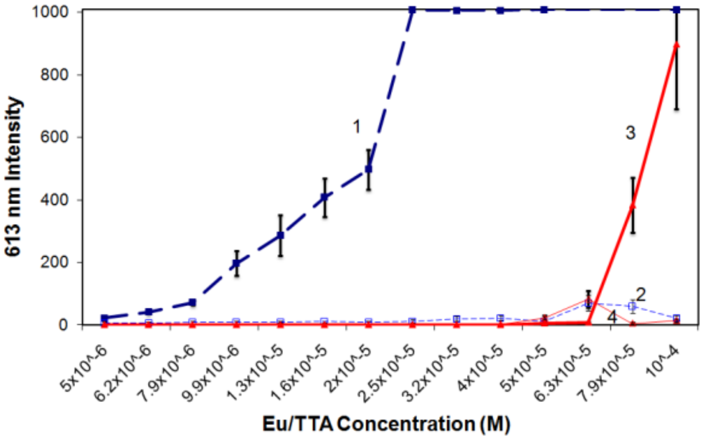

**FIGURE 6**. Eu/TTA 613-nm emission in (1) fresh water with NG, (2) fresh water without NG, (3) seawater with NG, and (4) seawater without NG. The reagent solvent (methanol) comprised 5% of the solution.

Studies with NaCl, $CaCl_2$, and HCl solutions confirmed that the potential adverse effects of low pH and alkaline earth metal ions extend to this detection method. Fig. 7 provides an example of the luminescence spectrometer results with the three different solutions. The NaCl solution allowed for much more fluorescence, while the $CaCl_2$ and HCl solutions severely quenched it. The Ca ion caused substantial quenching even though its concentration was half that of the Na ions. This, however, is not expected to create a big problem because neither the alkaline earth metal ion concentrations nor the pH are expected to vary significantly outside of that in the natural seawater that was used in the experiments herein[35].

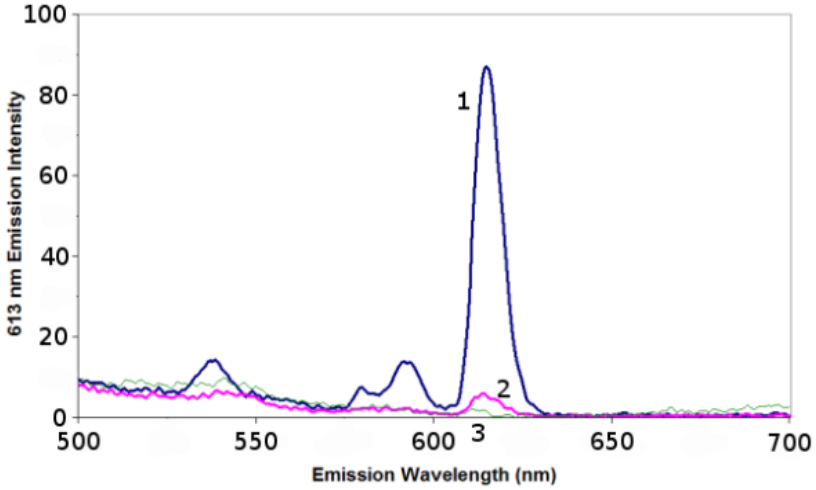

**FIGURE 7.** Eu/TTA ($1 \times 10^{-3}$ *M*) 613-nm emission in distilled water solutions of (1) NaCl, (2) $CaCl_2$, and (3) HCl. The reagent solvent (methanol) comprised 5% of the solution.

### Fluorometer Calibration to Determine an Appropriate Eu/TTA Concentration

Calibration of the fluorometer with blanks of seawater, reagent, and NG indicated that a Eu/TTA concentration of $4 \times 10^{-4}$ *M* provided excellent results. This concentration allowed for a strong fluorescence signal to the sensor, but did not saturate it. During this experimentation phase, it was also observed that the fluorescence oscillated at higher NG concentrations before stabilizing after a short time (<3 min). To account for this, a reaction time of 5 min was allowed. Based on the previously completed studies on the effects of the solvent, the reagent/methanol solution was adjusted to comprise 8% of the total solution. Fig. 8 shows the fluorometer calibration results.

### Flow-Through Configuration Demonstration

Using the customized fluorometer and static mixer, the sensing method was tested in a flowing configuration and NG was detected. Three solutions were evaluated in the flowing sensor: (1) seawater; (2) seawater and Eu/TTA ($1.75 \times 10^{-5}$ *M*); and (3) seawater, Eu/TTA ($1.75 \times 10^{-5}$ *M*), and NG ($5 \times 10^{-4}$ *M*). The NG was added by dissolving it in the seawater and the Eu/TTA solution was injected into the seawater stream with methanol at a mixing proportion of 8%. These three solutions resulted in fluorometer outputs as shown in Fig. 9.

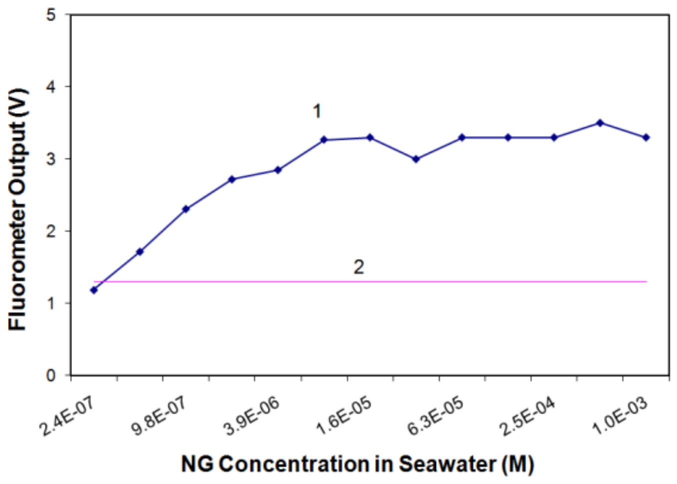

**FIGURE 8.** Fluorometer output of Eu/TTA (4 × $10^{-4}$ *M*)/seawater solution (5-min reaction time, 8% methanol) (1) with NG and (2) without NG.

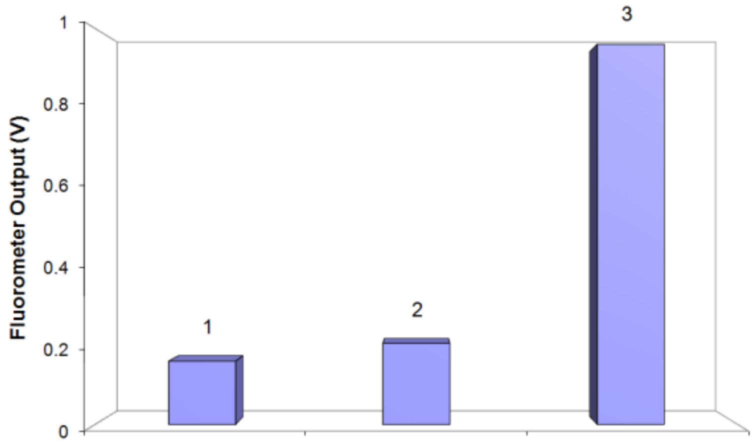

**FIGURE 9.** Flowing setup detector output. (1) Seawater only; (2) seawater and Eu/TTA (2 × $10^{-4}$ *M*); (3) seawater, NG (5 × $10^{-4}$ *M*), and Eu/TTA (2 × $10^{-4}$ *M*). The reagent solvent (methanol) comprised 8% of each solution that included Eu/TTA.

### DISCUSSION

From this research, it was determined that Eu/TTA compounds can be used to tag and detect waterborne explosive (NG) traces fluorescently. Similar to the dry detection methods described by Menzel et al.[2,3], this method relies on the aqueous quenching of Eu's fluorescence when NG is absent and its preservation when NG is present. However, this method is fundamentally different from Menzel et al.'s work because it is conducted in an aqueous medium. In the dry method, water is applied last, after the explosive trace and fluorescent compound have reacted, so that it quenches any *remaining* unreacted fluorescent compounds. Statistically speaking, in an aqueous solution, the Eu compounds are likely to contact water molecules before trace explosives, which would require the explosive compound to *overcome* the Eu/water bond so that it could attach itself to the Eu ion. By adapting this method to aqueous environments, it was clarified that the NG does indeed preferentially replace water on the Eu compound. Water-borne NG is able to recover Eu's fluorescent properties, even after it has already been quenched.

This likely occurs because NG's negatively charged nitrite moiety is more strongly attracted to the positively charged Eu ion's free bonding site than are water molecules. By reviewing NG's nitrite structure, it becomes apparent that it exhibits dipolar properties, similar to those seen with water molecules, that would make it attracted to the Eu ion. However, in addition to this, one of the oxygen atoms on the nitrite groups is connected through only one bond, leaving it with an additional negative charge. This charge likely makes the nitrite, and therefore the explosive group, more attracted to the free site on the cationic Eu ion than water.

Considering that this detection method targets the nitrite moiety of NG, it is also possible that it can be extended to other nitrite-bearing explosive types, such as RDX, Semtex, HMX, PETN, EGDN, nitrocellulose, tetryl, and Smokeless Power[30]. The structures of NG, RDX, and PETN are featured in Fig. 10 as examples to illustrate their common nitrite moieties. Menzel et al. have already demonstrated that the Eu/TTA compound can be used to detect RDX in their dry method[2]. However, further testing should be conducted to determine to which other explosive types this method may be extended. Additionally, the issue of false-positives should be evaluated. Other than through comparisons to similar trace explosive detection methods conducted in dry environments, it is not clear how susceptible this method is to false-positives, such as from fertilizers and environmental pollutants.

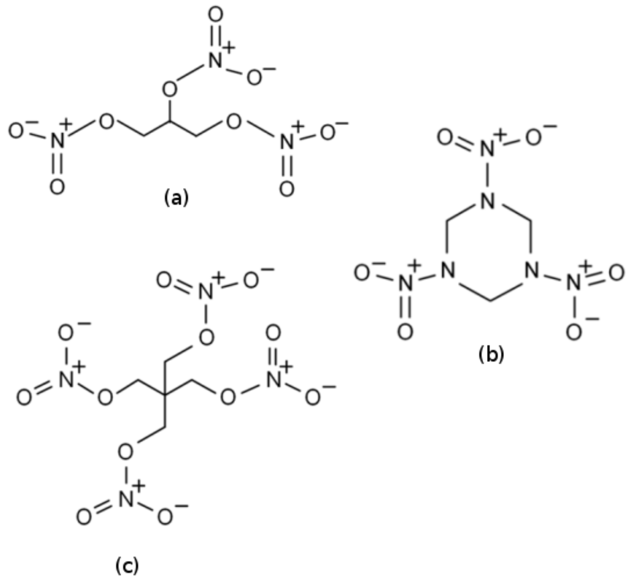

**FIGURE 10**. Explosive chemical structures: (a) NG, (b) RDX, (c) PETN.

Eu is capable of strong, long-lasting fluorescence, but in order to make use of this property, radiation-absorbent ligands must be attached to absorb and transfer energy to it. The type of ligand is important, as well as mixing order, if multiple ligands are used. For example, while Eu/TTA/OP and Eu/OP/TTA solutions contained the same components in the same ratios, their mixing orders were different and they consequently performed differently. It was also found that the Eu/TTA chelate produced significantly better results in underwater NG detection than Eu/TTA/OP and Eu/OP/TTA. The primary difference was that this compound was quenched by water when NG was absent, whereas the others were not. The lack of quenching observed when OP was used suggests that the OP ligand creates a hydrophobic environment around the Eu ion, with or without the presence of NG, thereby negating it as a possible sensitizing ligand for this application.

Ultimately, this method was designed for application in a portable, economical seawater sensor. Keeping this in mind, an effort was made to use sensitizing ligands that could be excited with standard LED wavelengths. TTA is optimally excited at 382 nm, but the output of a 370-nm LED was shown to suffice. OP on the other hand, required excitation at a much shorter wavelength (310 nm) that is not as easily attained with commercially available LEDs, providing another reason to exclude it from this effort.

Focusing on the development of a seawater sensor raised another issue in the potential interference from the various constituents found in seawater, vs. pure water. Alkaline earth metal ions (specifically calcium) and low pH conditions are known to interfere with sensitized lanthanide fluorescence. Alkaline earth metal ions are believed to support metal-exchange reactions with the lanthanide ions, leaving them without protective ligands and prone to water quenching[29,31]. Low pH adversely affects the energy transfer from the ligand to Eu, as well as reducing the chelate's stability. Acidic conditions cause the exchange reactions of the ligand to become more rapid and the effect of competing chelating agents may be increased[33]. These issues were confirmed herein by showing that both calcium and acid adversely affected the chemical method. This effect was also confirmed in another way by demonstrating that the method generally works better in pure water than in seawater. Although seawater performance was degraded somewhat, it is not expected to be a significant issue because the pH and alkaline earth metal concentrations in seawater from other locations are not expected to vary significantly from what was encountered with the natural seawater used herein. Furthermore, the pH problems with lanthanides have typically been reported to occur at lower levels than that normally found in seawater. Acid interference has been shown to occur below pH levels of 4 or 5[29,31,32,33], while the pH of natural seawater typically remains near 8[34,35,36]. While these agents somewhat degraded the chemical method performance in seawater, there is also a positive side: Detection is even better in freshwater, which could be useful for a number of applications, such as groundwater analysis. Using this method, NG was detected at 56 ppb in seawater. However, a specified concentration of NG ($10^{-3}$ *M*) could be detected in freshwater with less than 1/12 the amount of reagent required to detect the same amount in seawater.

The amount of the reagent solvent used (methanol) also affected the performance of the method through changes in solution clarity and fluorescence. Overall, less methanol led to better performance, but for practical reasons, the solvent amount can only be reduced so low. It is necessary to use enough solvent so that a steady stream of reagent can be injected into the seawater/water solution. The poor performance of solutions with high proportions of methanol may be explained by its low polarity. Being less polar than water, it allows constituents that are dissolved in water to come out of solution and collect together. Clarity reduction was observed most prominently in solutions containing NG. This lowered NG detectability in two ways: (1) reduced clarity hinders spectrometric analysis through diffraction, and (2) NG that precipitates out of solution and groups together is less available to react with Eu compounds.

Finally, in addition to developing the chemical method for use in aqueous solutions, this research demonstrated that the Eu/TTA trace explosive detection method can be implemented in a rapid, inexpensive, and field-deployable package. Such a sensor requires a relatively low solvent amount and can be run continuously in a flowing configuration, removing the need to intermittently collect and analyze samples. The particular drive for this research was the development of an underwater sensor that could be installed aboard an unmanned underwater vehicle (UUV), but many other applications could be envisioned in both sea- and freshwater.

### ACKNOWLEDGMENTS

The author would like to express sincere gratitude to Prof. Richard Granata for his advice and support. The support provided by the U.S. Office of Naval Research, Center for Coastline Security Technology, contract/PR No. N00014-05-C-0031 is also greatly appreciated.
